# Temporal trends of TAVI treatment characteristics in high volume centers in Germany 2013–2020

**DOI:** 10.1007/s00392-021-01963-3

**Published:** 2021-11-09

**Authors:** Victor Mauri, Mohamed Abdel-Wahab, Sabine Bleiziffer, Verena Veulemans, Alexander Sedaghat, Matti Adam, Georg Nickenig, Malte Kelm, Holger Thiele, Stephan Baldus, Tanja K. Rudolph

**Affiliations:** 1 Department of Cardiology, Faculty of Medicine, Heart Center, University of Cologne, Cologne, Germany; 2 Department of Cardiology, Heart Center Leipzig at University of Leipzig, Leipzig, Germany; 3 Department of Thoracic and Cardiovascular Surgery, Heart and Diabetes Center North Rhine-Westphalia, Ruhr-University Bochum, Bad Oeynhausen, Germany; 4 Department of Cardiology, Pulmonology and Vascular Medicine, Heart Center Düsseldorf, Heinrich Heine University, Medical Faculty, Düsseldorf, Germany; 5 Department of Medicine II, Heart Center Bonn, University Hospital Bonn, Bonn, Germany; 6 Department of General and Interventional Cardiology, Heart- and Diabetes Center North Rhine-Westphalia, Ruhr-University Bochum, Bad Oeynhausen, Germany

**Keywords:** TAVI, Low risk, Aortic stenosis

## Abstract

**Objective:**

To assess temporal trends of patient baseline characteristics, risk profile and outcome of transcatheter aortic valve implantation (TAVI) between 2013 and 2020.

**Background:**

Guideline recommendations and increasing confidence in TAVI therapy may have changed the selection of TAVI patients.

**Methods:**

Baseline risk profile and VARC-2 outcome of 15,344 patients undergoing TAVI at 5 high volume centers in Germany over the time period 2013–2020 was analyzed.

**Results:**

Over the 8 years, annual TAVI volumes more than doubled from 1071 in 2013 to 2996 in 2020. The baseline surgical risk estimated by the Society of Thoracic Surgeons (STS) score declined from 7.2 ± 6.2% to 4.6 ± 3.7% (*P* < 0.001) as a consequence of lower comorbidity burden, whereas mean age remained unchanged (2013 81.0 ± 6.1; 2020 80.8 ± 6.4; *P* = 0.976) with patients ≥ 80 years accounting for about two-third of the treated cohort.

Periprocedural complications including bleeding (2013 24.5%; 2020 12.1%; *P* < 0.001), vascular complications (2013 20.7%; 2020 11.7%; *P* < 0.001) and new permanent pacemaker implantation (2013 20.1%; 2020 13.8%, *P* < 0.001) decreased significantly. Similarly, the 30-day mortality decreased from 5.4% to 2.1% (*P* < 0.001), but remained high in high-risk patients (STS > 8% 2013 7.5%; 2020 6.9%; *P* = 0.778).

**Conclusion:**

From 2013 to 2020, mortality and burden of complications following TAVI procedure significantly decreased in a large multicenter registry from Germany. Proportion of elderly patients remained stable, while the surgical risk profile decreased.

**Graphical abstract:**

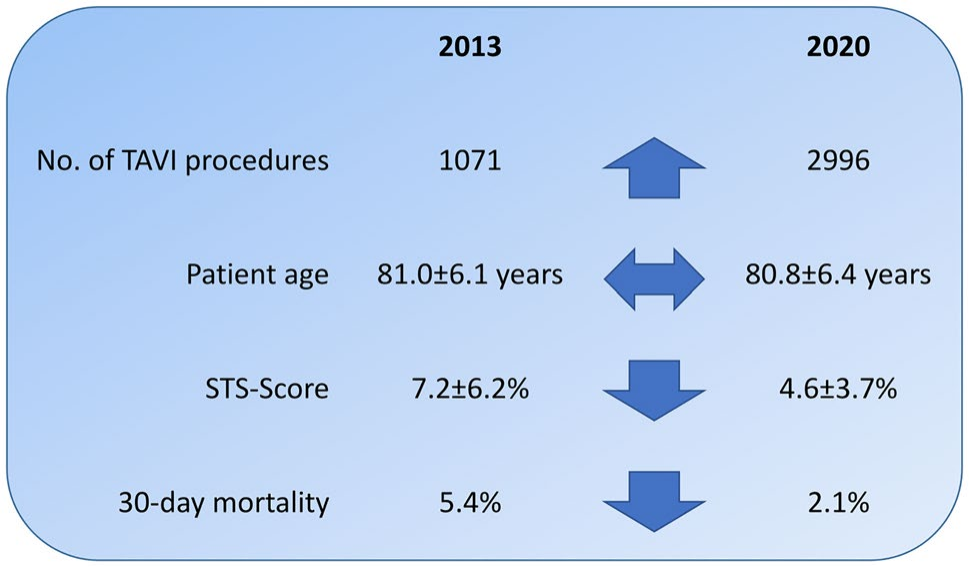

**Supplementary Information:**

The online version contains supplementary material available at 10.1007/s00392-021-01963-3.

## Introduction

Transcatheter aortic valve implantation (TAVI) has changed the treatment options for patients with symptomatic severe aortic stenosis fundamentally. TAVI was initially reserved for inoperable patients. Based on the evidence from the PARTNER 1A and CoreValve high-risk trial TAVI became the standard treatment option in patients with aortic stenosis and a high surgical risk based on the EuroSCORE or STS score [1, 2]. The ESC/EACTS guidelines for the management of valvular heart disease published in 2017 already recommended TAVI even in intermediate risk patients taking into account the results from the PARTNER II trial and the SURTAVI trial [3–5]. Since the EuroSCORE and STS score have been proven to perform poorly in TAVI populations, these guidelines suggest that the decision for TAVI should also be based on individual risk factors not covered by the scores and on the age of the patient [6]. Following these recommendations, the individual clinical judgement and patient age became increasingly important factors for the decision to undergo surgical or interventional treatment. Furthermore, the role of the heart team for individual decision making was clearly highlighted [3].

Due to growing expertise in implantation technique as well as higher safety and efficacy in the latest TAVI devices, complication rate significantly decreased over the last decade, whereas procedural success and outcome markedly improved [7, 8].

In this context, German national guidelines decreased the age limit, from initially 85 years to currently 75 years in low-risk patients, making the therapy potentially available to a broader patient collective [9].

However, it has not been investigated so far, whether the recommendations of the guidelines and/or the increasing confidence in TAVI therapy really impacts the selection and outcome of patients undergoing the procedure. We therefore sought to investigate the trends of baseline characteristics mainly focusing on age and surgical risk of a large all-comers patient cohort undergoing TAVI in five high volume TAVI centers in Germany from 2013 to 2020.

## Methods

All 15,344 consecutive patients undergoing TAVI for severe native aortic stenosis between January 2013 and December 2020 at 5 high volume centers in Germany (University Hospital Bonn, University Hospital Cologne, University Hospital Düsseldorf, Heart Center Leipzig at University of Leipzig, and Heart and Diabetes Center Bad Oeynhausen) were analyzed. Data were collected prospectively within the respective institutional registries and analyzed retrospectively with approval of the institutional review board of the respective academic center. The study complied with the Declaration of Helsinki. Central analysis was based on pseudonymized data. Eligibility of the individual candidate for TAVI was decided within the local institutional multidisciplinary heart team. Temporal trends of patient baseline characteristics as well as procedural outcomes and 30-day mortality were analyzed. Subgroups based on age (< 75 years; 75–80 years;  ≥ 80 years) and predicted surgical risk (STS-Score low: < 4%; intermediate 4–8%; high  ≥ 8%) were analyzed. Procedural outcomes were reported according to the VARC-2 consensus [10]. Risk-score calculation as well as clinical endpoints were site reported.

### Statistical analysis

Continuous variables are presented as mean ± standard deviation, categorical variables as frequencies and percentages. Analysis of categorical variables was performed with the Chi square or Fisher exact test. Continuous variables were analyzed with the Kruskal–Wallis test. Two-sided *P* values < 0.05 were considered statistically significant. All statistical analyses were performed with IBM SPSS Statistics, Version 27.

## Results

### Age and predicted surgical risk

**Fig. 1:**
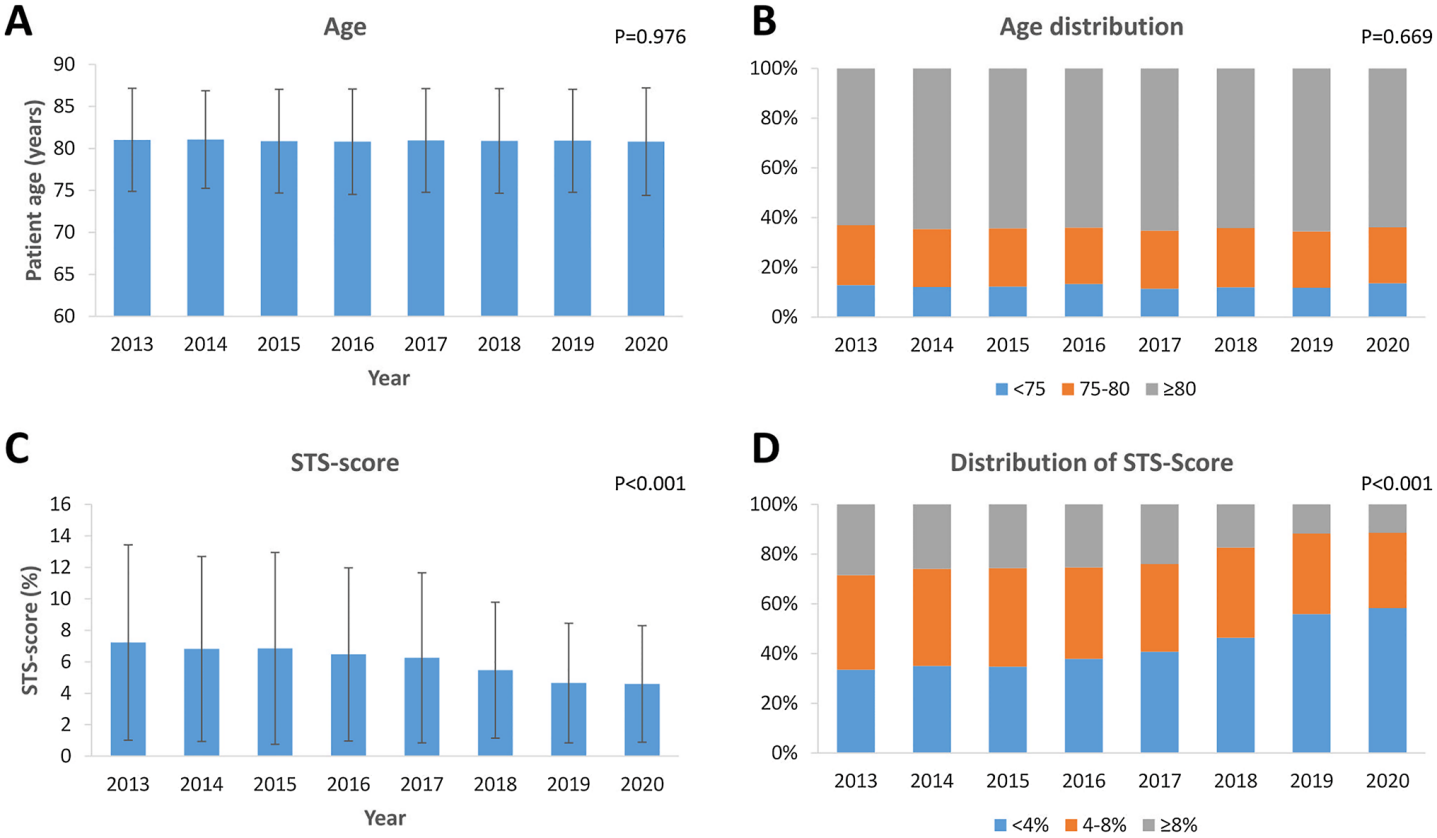
Temporal trends of **A**: mean patient age; **B**: distribution of age; **C**: predicted surgical risk (STS-Score); and **D**: risk categories by STS-score. Mean patient age remained stable, whereas mean STS-score declined significantly, driven by a growing proportion of low-risk patients

**Fig. 2:**
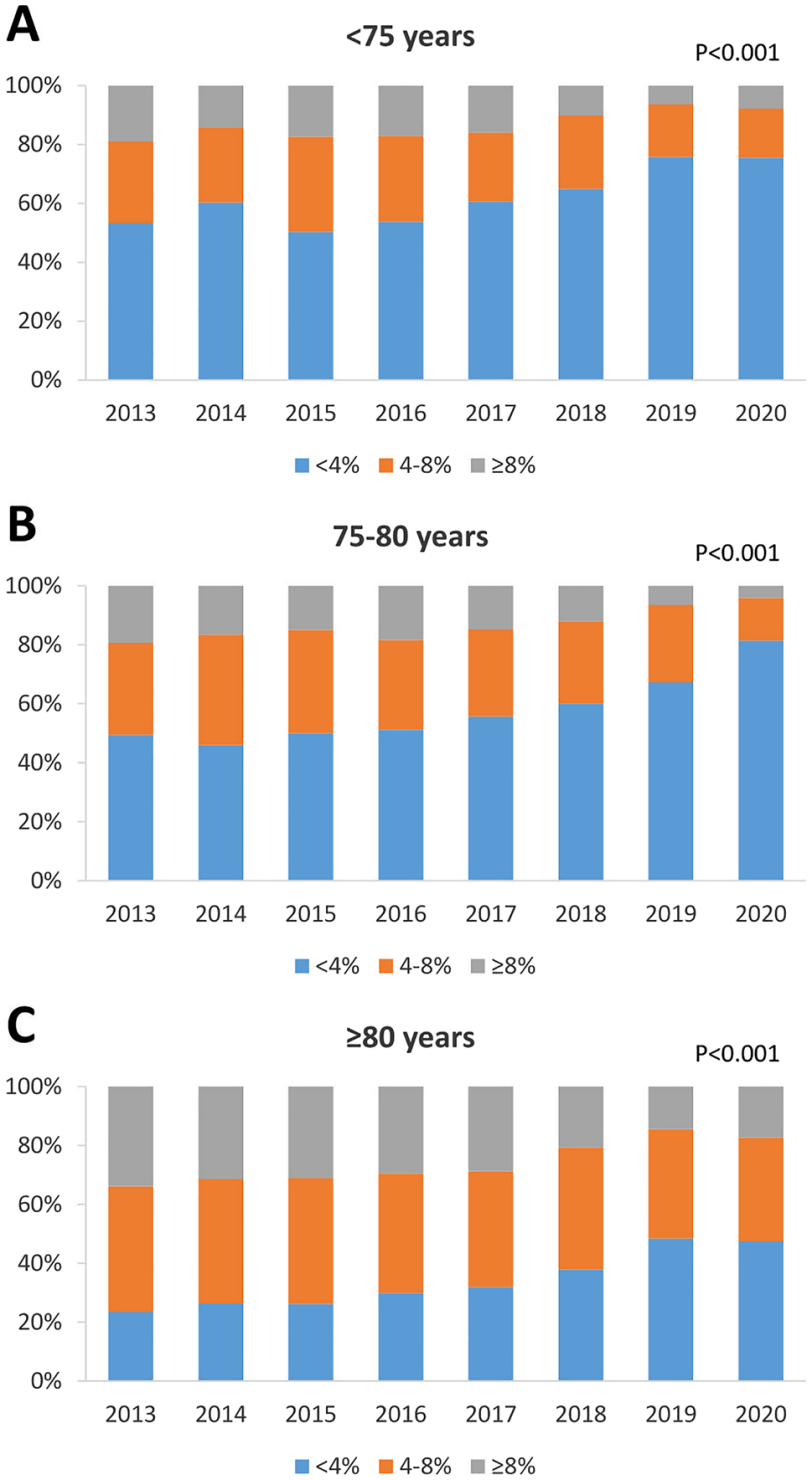
Temporal trends of risk stratification by STS-Score in the age groups **A**:  < 75 years; **B**: 75–80 years; **C**: > 80 years

In this analysis, a total of 15,344 patients undergoing TAVI from 2013 to 2020 were included. The annual number of patients treated with TAVI increased steadily from 1071 in 2013 to 2996 in 2020. Mean age remained stable during the observational period (mean age 81.0 ± 6.1 years; *P* = 0.680) as did the percentage of the pre-defined age groups (< 75 years, 75–80 years and ≥ 80 years). The main age group treated by TAVI was the group ≥ 80 years accounting for approximately 65% of all patients (Fig. 1A, B). The predicted surgical risk as assessed by the STS score (2013 7.2 ± 6.2%, 2020 4.6 ± 3.7%; *P* < 0.001) and the EuroScore II (2013 7.4 ± 6.7%, 2020 5.2 ± 5.3%; *P* < 0.001) steadily decreased from 2013 to 2020 (Fig. 1C). Looking at the three pre-defined STS score risk groups (low  < 4%, intermediate 4–8% and high  ≥ 8%) there was a significant increase of low-risk patients (2013 33.5% to 2020 58.0%; *P* < 0.001; Fig. 1D). Although the proportion of high-risk patients decreased, the absolute number remained stable over the study period. This finding was observed consistently in all pre-defined age groups (*P* < 0.001 for all; Fig. 2).

### Baseline patient characteristics

In line with decreasing predicted surgical risk, comorbidities of the analyzed TAVI patients changed over time. Accordingly, a significant decrease in diabetes mellitus, chronic obstructive pulmonary disease and previous cardiac surgery was observed. The percentage of patients with atrial fibrillation, coronary artery disease or previous stroke/TIA did not change in the observational period. A slight increase in previous myocardial infarction was observed. All baseline characteristics are shown in Table 1.

**Table 1:** Patient baseline characteristics by year

Year	2013	2014	2015	2016	2017	2018	2019	2020	*P* value
*N*	1071	1212	1512	1723	2208	2234	2388	2996	
Age (years)	81.0 ± 6.1	81.1 ± 5.8	80.9 ± 6.2	80.8 ± 6.3	80.9 ± 6.2	80.9 ± 6.2	80.9 ± 6.1	80.8 ± 6.4	0.976
< 75 years	137 (12.8)	146 (12.0)	185 (12.2)	229 (13.3)	251 (11.4)	268 (12.0)	281 (11.8)	408 (13.6)	0.669
75–80 years	259 (24.2)	283 (23.3)	354 (23.4)	391 (22.7)	516 (23.4)	532 (23.8)	541 (22.7)	673 (22.5)	
≥ 80 years	675 (63.0)	783 (64.6)	973 (64.4)	1103 (64.0)	1441 (65.3)	1434 (64.2)	1566 (65.6)	1915 (63.9)	
STS-Score (%)	7.2 ± 6.2	6.8 ± 5.9	6.8 ± 6.1	6.5 ± 5.5	6.2 ± 5.4	5.5 ± 4.3	4.6 ± 3.8	4.6 ± 3.7	< 0.001
< 4%	359 (33.5)	424 (35.0)	525 (34.7)	653 (37.9)	899 (40.7)	1036 (46.4)	1335 (55.9)	1737 (58.0)	< 0.001
4–8%	407 (38.0)	474 (39.1)	599 (39.6)	634 (36.8)	779 (35.3)	811 (36.3)	773 (32.4)	903 (30.1)	
≥ 8%	305 (28.5)	314 (25.9)	388 (25.7)	436 (25.3)	530 (24.0)	387 (17.3)	280 (11.7)	341 (11.4)	
EuroSCORE II (%)	7.4 ± 6.7	7.4 ± 7.4	7.8 ± 7.9	7.1 ± 7.1	6.7 ± 7.2	6.2 ± 6.3	5.4 ± 5.7	5.2 ± 5.3	< 0.001
Female sex	584 (54.5)	666 (55.0)	790 (52.2)	909 (52.8)	1140 (51.6)	1168 (52.3)	1170 (49.0)	1374 (45.9)	< 0.001
Hypertension	996 (93.0)	1118 (92.2)	1385 (91.7)	1393 (90.6)	2000 (90.6)	2003 (90.0)	2069 (86.9)	2600 (86.8)	< 0.001
Diabetes	408 (38.1)	439 (36.2)	521 (34.5)	498 (32.4)	787 (35.7)	755 (33.9)	775 (32.5)	1026 (34.2)	0.119
GFR (ml/min)	56 ± 21	56 ± 19	55 ± 21	55 ± 20	56 ± 20	56 ± 20	56 ± 19	57 ± 20	0.053
Dialysis	34 (3.2)	38 (3.1)	59 (4.4)	51 (3.3)	43 (2.0)	59 (2.7)	56 (2.4)	78 (2.7)	0.013
COPD	298 (31.1)	333 (29.4)	409 (29.1)	448 (27.0)	510 (25.2)	344 (15.7)	444 (19.6)	373 (18.8)	< 0.001
Peripheral artery disease	287 (26.8)	343 (28.3)	399 (26.4)	418 (27.2)	548 (24.8)	542 (24.4)	647 (27.2)	676 (22.6)	< 0.001
Coronary artery disease	588 (54.9)	645 (53.2)	722 (53.6)	931 (54.1)	1172 (53.1)	1411 (63.4)	1263 (53.1)	1851 (58.8)	< 0.001
Previous myocardial infarction	166 (15.5)	162 (13.4)	228 (15.1)	209 (13.6)	353 (16.0)	468 (21.0)	624 (26.3)	609 (20.3)	< 0.001
Previous cardiac surgery	216 (20.2)	234 (19.3)	301 (19.9)	208 (15.3)	224 (14.7)	237 (15.0)	316 (13.3)	389 (13.0)	< 0.001
Atrial fibrillation	428 (40.0)	485 (40.0)	588 (38.9)	657 (38.2)	852 (38.8)	945 (42.7)	934 (39.3)	1150 (38.4)	0.065
Previous stroke/TIA	131 (12.2)	147 (12.1)	193 (12.8)	176 (11.5)	163 (7.4)	278 (12.5)	240 (10.1)	316 (10.6)	< 0.001

Values are mean ± SD or *n* (%)

*COPD* chronic obstructive pulmonary disease, *GFR* glomerular filtration rate

### Procedural complications and outcome

**Fig. 3:**
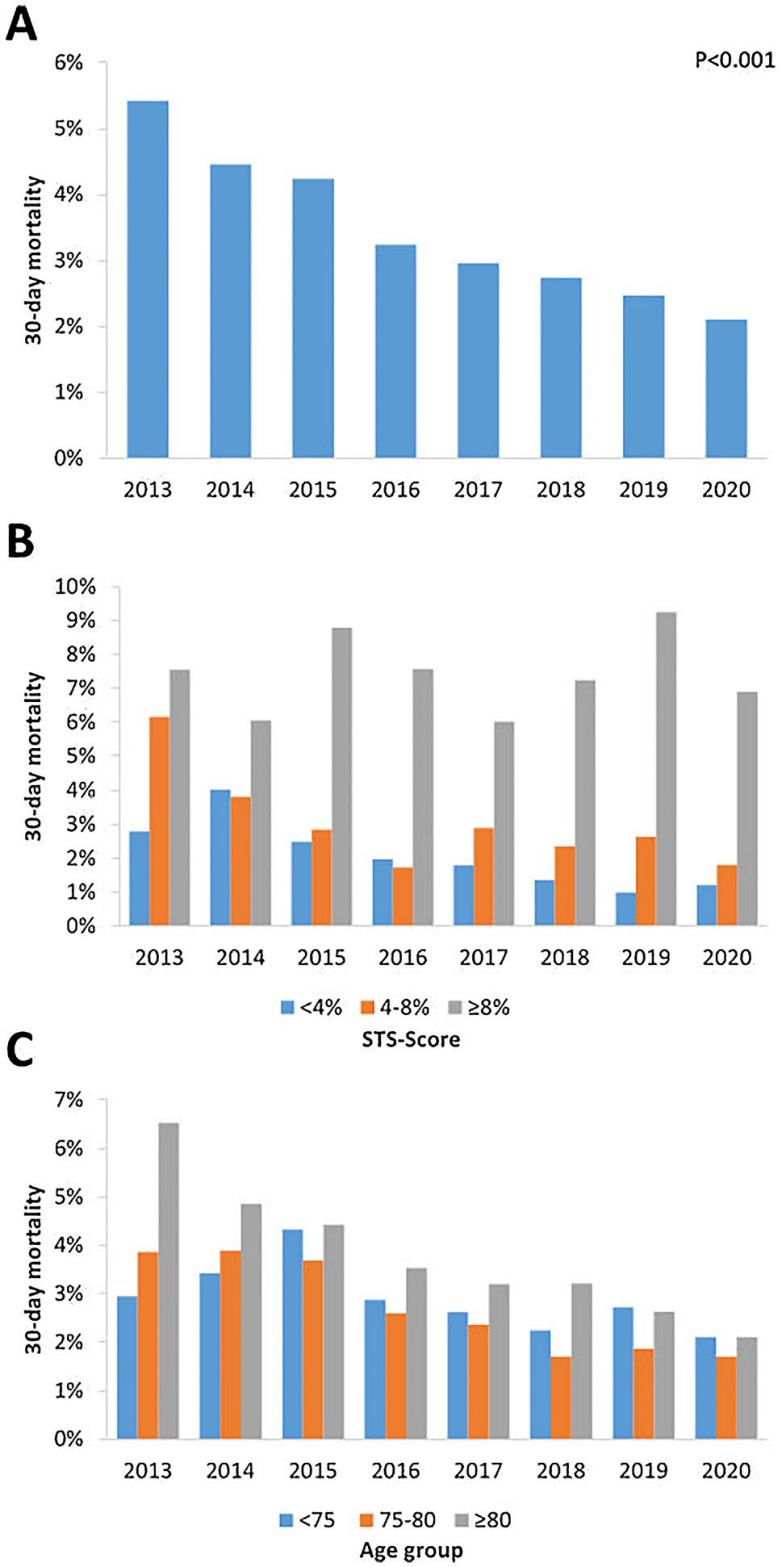
Trend of 30-day mortality. **A**: overall cohort (*P* < 0.001); *B*: according to STS-risk score group (< 4%: *P* = 0.001; 4–8%: *P* = 0.001; ≥ 8%: *P* = 0.778) *C*: according to age group (< 75 years: *P* = 0.949; 75–80: *P* = 0.167; ≥ 80: *P* < 0.001)

Procedural characteristics and complications are shown in Supplementary Table 1 and Table 2. Over the study period, there was a significant decline in bleeding (2013 23.5%; 2020 12.1%; *P* < 0.001) and vascular complications (2013 20.7%; 2020 11.7%; *P* < 0.001) as well as a reduction in the rate of new permanent pacemaker implantation (PPI; 2013 20.1%; 2020 13.8%, *P* < 0.001). The rare but severe complication of conversion to open heart surgery was reduced throughout the observational period (2013 5.5%; 2020 0.7%). Thirty-day mortality decreased significantly from initially 5.4–2.5% (*P* < 0.001; Table 2), which was mainly driven by a significantly decreasing 30-day mortality in patients > 80 years (2013 6.5%; 2020 2.1%; *P* < 0.001) and to a lesser extent in patients 75–80 years (2013 3.9%; 2020 1.7%, *P* = 0.325). Furthermore, 30-day mortality decreased significantly in low (STS < 4% 2013 2.8%; 2020 1.2%; *P* = 0.001) and intermediate risk patients (STS 4–8% 2013 6.2%; 2020 1.8%; *P* = 0.001), but remained high in high-risk patients (STS > 8% 20137.5%; 2020 6.9%; *P* = 0.778) (Fig. 3).

**Table 2:** Periprocedural complications by year

Year	2013	2014	2015	2016	2017	2018	2019	2020	*P* value
*N*	1071	1212	1512	1723	2208	2234	2388	2996	
Vascular complication	221 (20.7)	203 (16.7)	314 (20.8)	241 (14.0)	319 (14.5)	334 (15.8)	238 (12.2)	352 (11.7)	< 0.001
Major	121 (11.3)	72 (5.9)	65 (4.3)	48 (2.8)	77 (3.5)	73 (3.4)	52 (2.7)	64 (2.1)	< 0.001
Bleeding	251 (23.5)	245 (20.2)	320 (21.2)	148 (8.6)	224 (10.2)	270 (12.7)	195 (8.8)	362 (12.1)	< 0.001
Major/life-threatening	196 (18.3)	168 (13.9)	145 (9.6)	87 (5.1)	99 (4.5)	121 (5.7)	83 (3.7)	99 (3.3)	< 0.001
New permanent pacemaker	184 (20.1)	236 (22.2)	235 (18.5)	244 (16.3)	270 (14.0)	256 (13.2)	236 (12.6)	357 (13.8)	< 0.001
Stroke	38 (4.0)	29 (2.7)	45 (3.4)	43 (2.8)	36 (1.9)	35 (2.0)	46 (2.9)	78 (3.0)	0.017
Conversion to open surgery	30 (5.5)	59 (8.8)	55 (6.9)	75 (7.7)	49 (4.5)	39 (3.4)	28 (1.7)	8 (0.7)	< 0.001
In-hospital mortality	59 (5.5)	53 (4.4)	60 (4.0)	51 (3.0)	39 (1.8)	42 (2.0)	51 (2.1)	48 (1.6)	< 0.001
Thirty-day mortality	59 (5.5)	54 (4.5)	64 (4.2)	51 (3.2)	42 (3.0)	61 (2.7)	54 (2.5)	60 (2.1)	< 0.001
Thirty-day mortality by age									
< 75 years	4 (2.9)	5 (3.4)	8 (4.3)	6 (2.9)	4 (2.6)	6 (2.2)	7 (2.7)	10 (2.1)	0.949
75–80 years	10 (3.9)	11 (3.9)	13 (3.7)	9 (2.6)	7 (2.4)	9 (1.7)	9 (1.9)	11 (1.7)	0.167
≥ 80 years	44 (6.5)	38 (4.9)	43 (4.4)	36 (3.5)	31 (3.2)	46 (3.2)	38 (2.6)	39 (2.1)	< 0.001
Thirty-day mortality by STS-score									
< 4%	10 (2.8)	17 (4.0)	13 (2.5)	12 (2.0)	12 (1.8)	14 (1.4)	12 (1.0)	21 (1.2)	0.001
4–8%	25 (6.2)	18 (3.8)	17 (2.8)	10 (1.7)	14 (2.9)	19 (2.3)	19 (2.6)	16 (1.8)	0.001
≥ 8%	23 (7.5)	19 (6.1)	34 (8.8)	29 (7.6)	16 (6.0)	28 (7.2)	23 (9.2)	23 (6.9)	0.778

Values are *n* (%)

## Discussion

The present study comprises the analysis of trends in baseline characteristics and procedural outcome in all-comer TAVI patients from five high-volume centers in Germany over a time period of 8 years, representing approximately 10% of the German TAVI procedures. The main findings are (1) The mean age of patients receiving TAVI is still over 80 years; (2) There is a clear trend to treat lower risk patients with TAVI with accordingly less comorbidities; (3) procedural complications and 30-day mortality decreased significantly.

The numbers of TAVI procedures performed increased steadily in the five TAVI centers studied herein, which has also been observed in other large registries [11–15]. With a mean age of 81 years the investigated cohort is – with regard to age—more comparable to patients enrolled in the high and intermediate risk studies than to the recent randomized low-risk trials with a substantially lower mean age of 73 and 74 years, respectively [16, 17]. Only the NOTION trial studied elderly low-risk patients with a mean age of approximately 79 years [18]. Surprisingly, the distribution of the patients according to their age remained almost unchanged over the observation period. The rate of patients with an age between 75 and 80 years was constantly around 22%. At the beginning of 2020 the German national recommendations, endorsed by the German Societies of Cardiology and Cardio-thoracic surgery, recommended TAVI as first-line therapy in this age category [9]. The data of present analysis were collected until December 2020, so the uptake of this recommendation in clinical practice might not yet be fully reflected. The group of patients below 75 years receiving TAVI was constantly around 10%. Of interest, at the beginning of the observational period the STS score of this group was significantly higher as compared to the more recent period. This might be explained by the fact that the decision to treat these relatively young patients with TAVI has initially been mainly driven by their surgical risk and their comorbidities. Whether factors not covered by the applied risk scores (e.g., porcelain aorta or sequelae of radiation) or more liberal indication for TAVI are responsible for the observed shift cannot be answered from our data.

The majority of patients (> 60%) was over 80 years, which is comparable to other registry data [12–15, 19]. Since symptomatic severe aortic stenosis mostly occurs in the late seventh or eighth life decade, there are fewer young patients who need to undergo TAVI/SAVR which might also contribute to the observed age distribution. Furthermore, younger patients might also more often be treated surgically as they present frequently with anatomies less favorable for TAVI (for example bicuspid valves) or additional pathologies (e.g., coronary artery disease, multivalve disease, aortopathy) as their older counterparts.

The surgical risk based on the STS score overall reflects an intermediate risk cohort in our study. However, the mean STS score declined significantly during the observation period from 7.2% in 2013 to 4.6% in 2020. The proportion of low-risk and intermediate-risk patients increased steadily whereas the percentage of high-risk patients decreased. In 2020, more than 50% of the treated patients were classified as low-risk patients and less than 20% of the patients were classified as high-risk patients.

Our data reinforce the observation that recommendations of 2017 ESC/EACTS guidelines have been followed in clinical practice to prefer TAVI over SAVR in patients with increased surgical risk (STS score > 4) and in the elderly (> 80/85 years) independent of their surgical risk [3].

The increase of low-risk patients in the age group < 75 and 75–80 years is mainly pronounced in 2019 and 2020, which might be explained by a change in clinical practice probably based on the results of the Evolut and PARTNER 3 low-risk trials showing non-inferiority or even superiority of TAVI over SAVR [16, 17]. Since these two groups made up for only 30% of the overall cohort, the impact of this change on age distribution is currently rather small. Whether a potential change in guideline recommendations leads to a more pronounced shift in the treated patient population needs to be investigated in the future.

The rate of vascular complications and bleeding events decreased significantly over time and is comparable to data from other studies [13, 19]. Improvements and downsizing in vascular sheaths for almost all currently available TAVI devices are contributing to this finding as does the increasing operators’ experience. The PPI rate declined significantly in our population, however there are conflicting data within the literature with some studies even showing increasing rates of PPI over time [14, 20–22]. Several factors including baseline conduction disturbances, sizing, implantation depth, membranous septum length, and calcium distribution have been identified as important determining factors of the subsequent need for pacemaker implantation [23–25]. Accordingly, changes in implantation technique, namely cusp-overlap for self-expanding TAVI, and an individualized TAVI device selection have been progressively adopted in clinical practice in the participating centers over the recent years, possibly resulting in declining pacemaker rates in the current analysis. In addition to that, low-risk patients have been shown to have a lower risk for conduction disturbances requiring new PPI [22].

Thirty-day mortality declined significantly over the observational period, a phenomenon which has been reported elsewhere [12–14]. The decline was mainly observed in the patient group > 80 years and in intermediate risk patients. Operators’ experience and a more standardized less invasive procedure might be the main contributors to this observation. Of note, in high-risk patients the 30-day mortality rate remained almost unchanged high at approximately 8%, which is an important information for patient selection and patient counselling in daily clinical practice.

In low- and intermediate-risk patients, 30-day mortality in this all-comer patient population was higher as in randomized trials and basically unchanged over the last 5 years. In contrast to the highly selected patients within randomized trials, an all-comers population was included in this study that more likely represents real-world practice.

Thirty-day mortality in the group of 75–80 years declined significantly and was stable at about 2% throughout the last 3 years. Of interest, 30-day mortality in patients < 75 years is higher (approximately 3%) as compared to the group of 75–80 years, as is the proportion of patients with an STS score ≥ 8. Consequently, in this relatively young patient group the decision for TAVI seems to be based on high surgical risk or other issues not covered by the risk scores and not a sign of increased use of TAVI in younger patients.

## Limitations

This is an observational analysis with all its inherent limitations. All parameters were site reported and only German sites were included. Detailed information on patient selection criteria from each center were not available.

## Conclusions

This analysis provides important insights into current clinical practice. In five high volume centers, from 2013 to 2020, there has been a relevant expansion of TAVI procedures towards patients with lower surgical risk despite advanced age, which remained > 80 years over time. Elderly patients can be treated safely with TAVI irrespective of their predicted surgical risk. The often-expressed criticism of an unjustified expansion of TAVI indications towards young low-risk patients cannot be confirmed from this data.

## Supplementary Information

Below is the link to the electronic supplementary material.

Supplementary file1 (DOCX 24 KB)
