# Supplementary material for: Temporal trends of TAVI treatment characteristics in high volume centers in Germany 2013–2020
Source: Clin Res Cardiol. 2021 Nov 9;111(8):881–8. doi: 10.1007/s00392-021-01963-3 (PMC9334359; doi:10.1007/s00392-021-01963-3)
Supplement: Supplementary file 1 — Supplementary file1 (DOCX 24 KB) [file 392_2021_1963_MOESM1_ESM.docx]

### Supplementary Table 1: Procedural characteristics

|  |  |  |  |  |  |  |
| --- | --- | --- | --- | --- | --- | --- |
| **Valve type** |  |  |  |  |  |  |
| SAPIEN | 577 | (3,8) |  |  |  |  |
| SAPIEN 3/ultra | 5069 | (33.0) |  |  |  |  |
| CoreValve | 882 | (5.7) |  |  |  |  |
| CoreValve Evolut R/Pro | 5610 | (36.6) |  |  |  |  |
| Accurate neo/neo2 | 1308 | (8.5) |  |  |  |  |
| Lotus/Lotus edge | 306 | (2.0) |  |  |  |  |
| Others | 430 | (2.8) |  |  |  |  |
|  | 59 | (5.5) |  |  |  |  |
|  | 59 | (5.5) |  |  |  |  |
| **Access** | | | |  |  |  |
| transfemoral | 12973 | (91.5) |  |  |  |  |
| transapical | 1041 | (7.3) |  |  |  |  |
| other | 166 | (1.2) |  |  |  |  |

Values are n (%).
